# Drowning and Submersion Deaths in Bathtubs and Associated Factors: A Descriptive and Ecological Study in Japan, 1995–2020

**DOI:** 10.2188/jea.JE20250032

**Published:** 2025-11-05

**Authors:** Yoshiaki Tai, Kenji Obayashi, Yuki Yamagami, Keigo Saeki

**Affiliations:** Department of Epidemiology, Nara Medical University School of Medicine, Nara, Japan

**Keywords:** drowning, hot tub bathing, bath-related death, older adults

## Abstract

**Background:**

Older Japanese adults have the highest drowning mortality rates globally, likely due to in-home bathing customs. However, epidemiological evidence of preventive strategies based on national data is lacking. We aimed to describe the trends in bathtub drowning deaths (International Classification of Diseases, Tenth Revision code: W65) across Japan and explore factors that may reduce W65-coded deaths.

**Methods:**

We collected the data of all W65-coded deaths that occurred at home from 1995 to 2020 using death certificates from the Ministry of Health, Labour and Welfare. The national age-adjusted mortality rates (AMRs) and prefecture-specific age-standardized mortality ratios (SMRs) were calculated. Data on demographic, socioeconomic, environmental factors, and nursing care services were obtained from the Japan Portal Site of Official Statistics. Mixed-effects analysis was used to examine the association between SMR and potential contributing factors at the prefecture level.

**Results:**

We identified 99,930 W65-coded deaths at home, with the highest incidence among individuals aged 80–84 years, peaking in January. Since 2010, AMRs have consistently exceeded 3.0 per 100,000. An inverse association was found between SMR and the number of geriatric health service facilities and senior welfare centers per capita (coefficients per 1 standard deviation increase, −0.09; 95% confidence interval (CI), −0.13 to −0.05, *P* < 0.001 and −0.07; 95% CI, −0.11 to −0.02, *P* = 0.004, respectively), after adjusting for demographic, socioeconomic, and environmental factors.

**Conclusion:**

Sustained high AMRs suggest that the rising death toll was not solely due to aging. Increased access to nursing care facilities may help prevent W65-coded deaths.

## INTRODUCTION

Drowning is a prevalent cause of accidental death and a significant contributor to childhood mortality worldwide.^1^ Low- and middle-income nations exhibit the highest drowning rates, constituting over 90% of global incidents.^2^ Global mortality due to unintentional drowning has decreased by approximately 57% from 1990 to 2017.^1^ In contrast, Japan presents a different pattern. Older Japanese individuals, representing the primary demographic of domestic drowning deaths, have the highest drowning mortality rates globally,^3^ primarily because of the custom of hot-tub bathing at home. Deaths from unintentional drowning and submersion in bathtubs (International Classification of Diseases, 10th Revision [ICD-10] code: W65) have been increasing, ranging between 4,000 and 6,000 deaths annually over the past 2 decades and have exceeded deaths due to all causes of traffic accidents (ICD-10 codes: V01–V98) since 2016 in Japan.

Hot-tub bathing is a traditional Japanese practice that promotes cleanliness and comfort. Typically, individuals soak in tubs deep enough to submerge up to their neck while reclining,^4^ with water temperatures of around 41°C.^5^ Hot-tub bathing has been associated with health benefits, such as lower night-time blood pressure,^5^ improved sleep quality,^6^ reduced prevalence of depression,^7^ and decreased risk of cardiovascular disease.^8^ However, bath-related deaths, sudden deaths occurring during bathing, are a significant public health challenge in Japan. These deaths encompass exogenous causes, including drowning, intoxication, and heat illness, as well as endogenous causes, such as sudden cardiovascular disease, stroke, and epilepsy. In Japan, bath-related deaths are estimated to be over 18,000 per year and are projected to exceed 27,000 by 2035 due to the aging population.^9^^,^^10^ Previous studies have explored potential mechanisms and mitigation strategies from the perspectives of physiology,^11^ emergency medicine,^9^ and forensic medicine.^12^ However, due to difficulties in determining etiologies, such as lack of witnesses,^13^ complex mechanisms,^14^ cases without signs of water aspiration, and low autopsy rates,^15^ the implementation of effective preventive measures remains challenging.

Epidemiological evidence shows trends in bath-related deaths by age, sex, season, and outdoor temperature.^9^^,^^15^^–^^17^ However, this evidence is based on surveys from one or up to three prefectures. Furthermore, to the best of our knowledge, no study has conducted a comprehensive epidemiological investigation using the complete enumeration of bath-related deaths in Japan. Moreover, previous studies have neither examined the regional differences in bath-related mortality across all prefectures in Japan, nor the associations between bath-related mortality and factors, such as bathing environment, healthcare facilities, and financial conditions, at the prefectural level.

Thus, to examine the annual and regional trends of bath-related deaths in Japan, we collected census data on W65-coded cases occurring at home, which is the predominant classification for bath-related deaths (94.3% of deaths occurring at home and 79.1% of autopsied cases showing signs of water inhalation),^14^^,^^17^ computed nationwide age-adjusted mortality rates (AMRs), and prefecture-specific age-standardized mortality ratios (SMRs) from 1995 to 2020. Furthermore, we explored the factors associated with prefecture-level SMRs to guide preventative initiatives.

## METHODS

### Deaths from unintentional drowning and submersion in a bathtub (ICD-10 code W65) that occurred at home

We obtained data on all deaths attributed to ICD-10 code W65 in Japan from 1995 to 2020 using death certificate information provided by the National Vital Statistics, accessed under Article 33 of the Statistics Act of Japan through the Ministry of Health, Labour and Welfare. We excluded unintentional drowning and submersion cases prior to 1995 because Japan utilized ICD-9 coding at that time. From the death certificates, we requested data on the decedent’s age, sex, date of death, notifying prefecture, and the specific place of occurrence code for deaths from external causes. Using this place-of-occurrence code, we identified W65-coded cases that occurred at home.

### Calculation of AMRs and SMRs for W65 cases occurring at home

Population data from 1995 to 2020 were sourced from the Japan Census, conducted at 5-year intervals, with estimates provided for the intervening years. These data were categorized into 5-year age groups and prefectures. The oldest age groups were defined as those aged 80 years or older from 1995 to 2006 and 85 years or older from 2007 to 2020, following the age intervals established by the Japanese Census data. The 2015 Japanese population model was used as the standard population for age adjustment.^18^ Using these population figures, along with data on the incidence of W65-coded cases by age group and reporting prefecture, we calculated the annual AMRs for Japan, as well as the prefecture-specific annual SMRs for the period between 1995 and 2020.

### Prefecture-specific factors

Demographic, socioeconomic, and environmental data, along with nursing care service information, were retrieved from the Portal Site of Official Statistics of Japan (e-Stat).^19^ Demographic factors included the population density (persons/km^2^), percentage of single-person households with individuals aged ≥65 years, and percentage of individuals aged ≥65 years living alone. Socioeconomic indicators comprised social welfare expenses for older adults per individual aged ≥65 years (Japanese Yen [JPY]/person), as well as the employment rate of individuals aged ≥65 years. Environmental factors accounted for the lowest mean daytime outdoor temperature in the coldest month, highest mean daytime outdoor temperature in the hottest month, and annual number of snowfall days per prefecture.

In addition, we collected data on bathing-related factors, such as the number of public baths, home bathing service users, daycare service users, and senior welfare centers. In Japan, daycare services provide bathing assistance using specially designed tubs, whereas senior welfare centers typically offer affordable public baths and promote health benefits for the elderly. Furthermore, we gathered data on the number of health care facilities, including hospitals, nursing and medical facilities, and geriatric health service facilities. The number of healthcare facilities was standardized as the number per 100,000 people aged ≥65 years.

The annual alcohol consumption per capita (kiloliters/person) by prefecture was sourced from the statistics of the National Tax Agency of Japan.^20^ The mean altitude of residential areas in each prefectural capital was obtained from the Center for Spatial Information Science, University of Tokyo.^21^ The National Police Agency of Japan provided the number of corpse-handling cases by police, judicial autopsy cases, and other autopsy (administrative autopsy, consent autopsy, and autopsy based on the Death Investigation and Identification Act) cases. We calculated the proportion of judicial and other autopsy cases among corpse-handling cases.

This study was conducted in accordance with the principles outlined in the Declaration of Helsinki and approved by the Ethics Committee of Nara Medical University (approval no. 3557).

### Statistical analysis

Descriptive statistics for normally distributed continuous variables were presented as means and standard deviations (SDs), while categorical variables were summarized as counts and percentages. To illustrate the annual trend in W65 deaths and assess whether the recent increase in W65 deaths is attributable to Japan’s aging population, the annual number of W65 cases occurring at home and their AMRs were presented in bar and line charts, respectively. Additionally, to examine the monthly trends of W65-coded cases and determine whether the seasonal variations reported in previous studies aligned with our findings from the complete enumeration, a bar chart depicting the monthly distribution of W65-coded deaths from 1995 to 2020 was used. Similarly, the number of W65-coded deaths by 5-year age groups and sex was visualized using bar charts.

Geographical trends in the SMRs of W65-coded deaths across Japan’s prefectures were visualized using a color-coded map, where lighter colors indicated higher values. The prefectures with the highest SMRs in each traditional region were labeled and marked with arrows for clarity.

Additionally, bubble charts illustrated the association between SMR from W65, social welfare expenses for older adults per individual aged ≥65 years, and the proportion of autopsies among police-handled corpses, with the population of each prefecture represented by the bubble size. On the chart, we highlighted the prefectures that have and used to have cities with a medical examiner system, which may affect the accuracy and preference of W65 diagnosis. Using a linear regression model, we examined the association between prefecture-specific factors and SMR in 2020. These factors included the demographic (population density [persons/km^2^] and proportion of elderly single-person households [%]), socioeconomic (social welfare expense for individuals aged ≥65 years per capita [JPY/person], the proportion of employed individuals aged ≥65 years [%]), lifestyle (annual alcohol consumption per capita [kiloliter/person]), environmental (the mean of lowest daytime outdoor temperature in the coldest month [°C], number of days with snowfalls [days], and mean altitude of prefectural capital [meter]), and diagnostic factors (the proportions of judicial and the other forms of autopsies [%]).

The consistency of SMR and the factors related to bathing practice (the number of public baths, senior welfare centers, daycare service users, home bathing service users, nursing and medical facilities, and geriatric health service facilities per 100,000 people aged ≥65 years) within prefectures were assessed using intraclass correlation coefficients (ICCs) with a two-way mixed-effects model for consistency. We employed a linear mixed-effects model with a random intercept for each prefecture to investigate the association between prefecture-specific SMRs and the above-mentioned factors related to bathing practices. The variance-covariance matrices were specified to be unstructured. The model parameters were estimated using the restricted maximum likelihood. The adjusted model includes demographic, social, lifestyle, and environmental factors for each year in each prefecture. However, the mean altitude of the prefectural capital, proportion of employed individuals aged ≥65 years, proportion of elderly single-person households, and proportion of individuals ≥65 years living alone were excluded as covariates due to the lack of available data, which covered only 1, 5, 6, and 6 years between 1995 and 2020, respectively. The mean maximum daily temperature in the hottest month was excluded as a covariate due to its correlation with the mean minimum daily temperature in the coldest month.

For sensitivity analysis, we reanalyzed the data excluding 2020 to account for potential changes in bathing behaviors and bath-related deaths at home during the coronavirus disease 2019 pandemic. Geographical trends in the SMRs of W65-coded deaths in 2019 and a bubble chart illustrating the association between social welfare expenditure and SMRs in 2019 are presented. Similarly, the association between prefecture-level representative values and SMRs in 2019 is shown. Additionally, we applied the previously described linear mixed-effects model to data spanning from 1995 to 2019.

All statistical analyses and visualizations were performed using R software, version 4.3.3 (R Foundation for Statistical Computing, Vienna, Austria). Visualization of the Japanese map, intraclass correlation, and mixed-effect model were performed using the R packages rnaturalearth, ICC, and lmerTest, respectively. All *P*-values were derived from two-sided tests, and those less than 0.05 were considered statistically significant.

## RESULTS

A total of 111,063 W65-coded deaths were identified from the death certificate dataset between 1995 and 2020. Among these, 99,930 cases (90.0%) occurred in the “home” setting, 6,955 cases (6.3%) were in “trade and service areas,” 995 cases (0.9%) in “residential institutions,” 529 cases (0.5%) in “schools, other institutions, and public administrative places,” and 2,566 cases (2.3%) were recorded in “unspecified locations.” Notably, two cases had missing data on age; however, there were no missing data for sex, date of death, prefecture of report, or place of occurrence for external causes of death.

**Figure 1.**
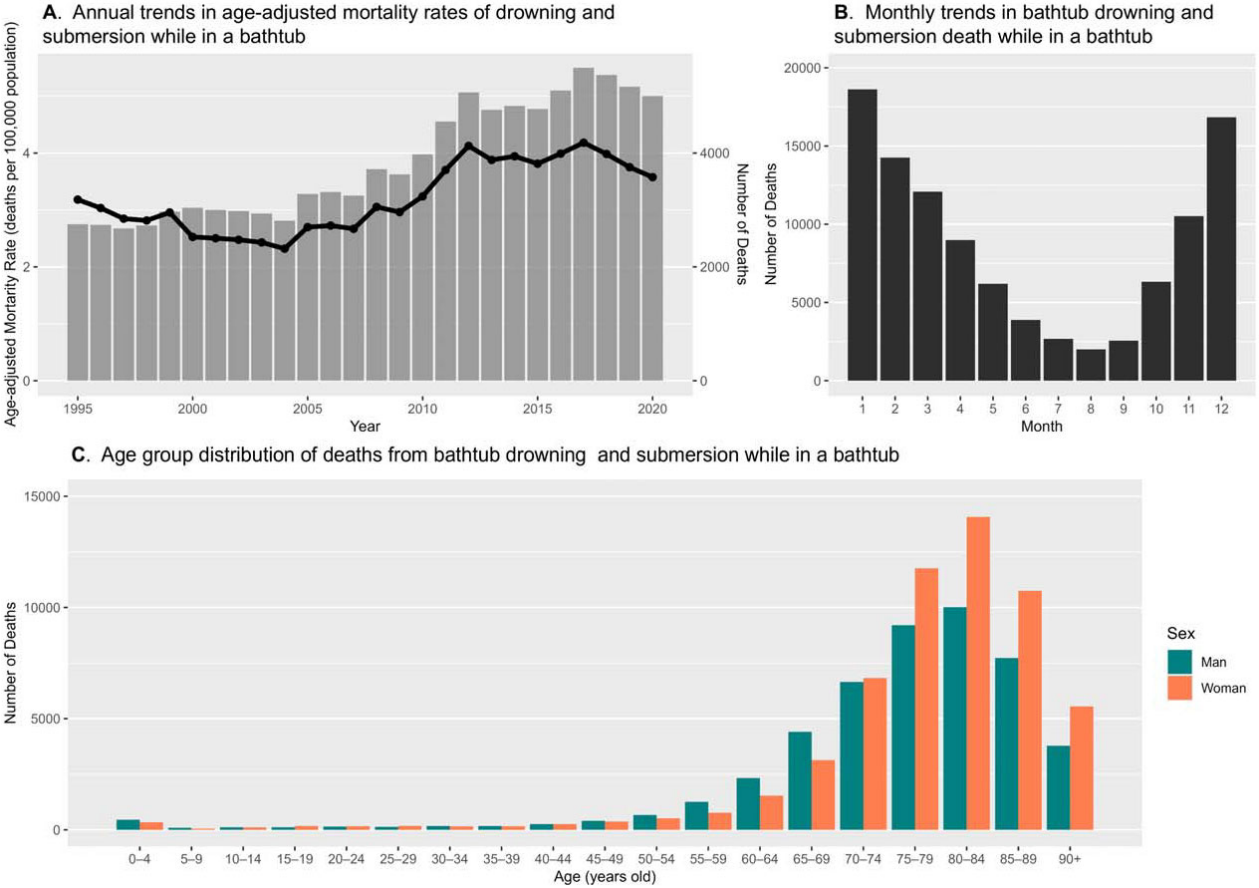
Trends in unintentional drowning and submersion while in bathtubs by year, month, and age group (1995–2020). (**A**) shows annual trends in the number of deaths and age-adjusted mortality rates from accidental drowning and submersion in a bathtub (International Classification of Diseases, Tenth Revision code, W65) at home, using a bar chart and line graph, respectively. (**B**) shows monthly trends in W65-coded deaths that occurred at home. (**C**) shows the age group distribution of W65-coded deaths that occurred at home.

Figure 1A illustrates the annual trends in the death toll and national AMRs for W65 at home. The number of at-home W65-coded deaths first exceeded 4,000 in 2011 and has consistently remained above this level since. Alterations in the national AMR of at-home W65 have paralleled the number of deaths since 2000. The months with the highest incidence of W65-coded deaths at home were January, February, and December, recording 17,774, 15,983, and 13,659 deaths, respectively (Figure 1B). These values were approximately 9.3, 8.4, and 7.2 times greater than the 1,901 instances recorded in August. The age ranges of 80–84 years and 75–79 years exhibit the highest and second-highest incidences of W65-coded deaths at home, respectively, for both sexes (Figure 1C). The percentage of W65-coded deaths occurring at home among those aged 65 years and older was 89.2% (*n* = 89,179).

**Figure 2.**
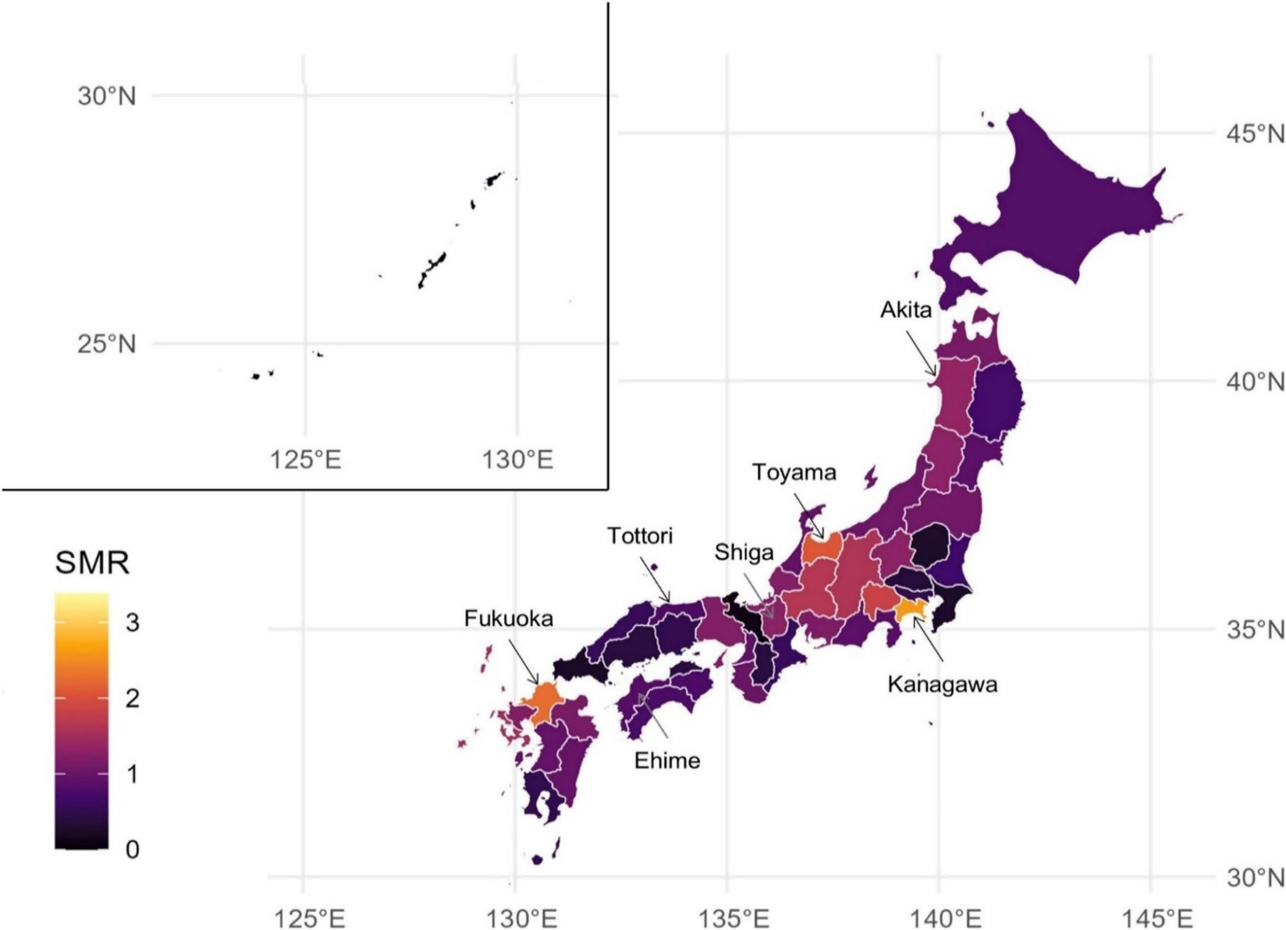
SMR of drowning and submersion in bathtubs by prefecture on the Japanese map in 2020. SMR refers to the prefecture-specific age-standardized mortality ratio for deaths classified under the International Classification of Diseases 10^th^ revision code W65 occurring at home.

Figure 2 depicts the geographical trends in SMRs for 2020. Alongside the notably elevated SMRs in Kanagawa and Fukuoka Prefectures, the Chubu region (particularly Toyama Prefecture) demonstrated comparatively high SMRs. These prefectures showed high SMRs in 2019 as well (eFigure 1). However, several prefectures in northern Japan, including Hokkaido and Aomori, did not exhibit elevated mortality rates. Kanagawa and Fukuoka Prefectures showed consistently elevated SMRs annually (eFigure 2). Conversely, the annual SMRs in Okinawa Prefecture, located in the southernmost region of Japan, remained the lowest from 1995 to 2020.

**Figure 3.**
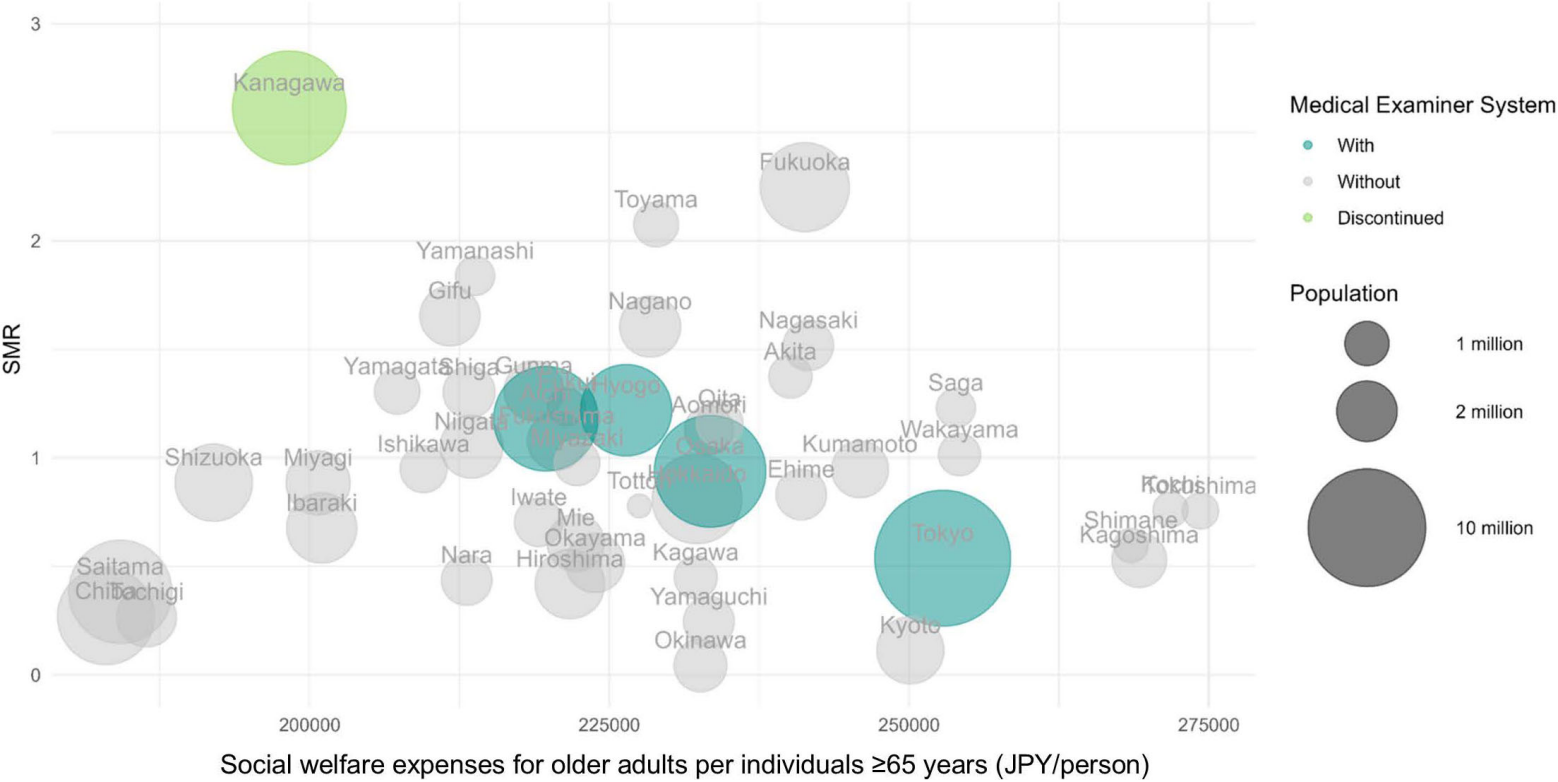
Association of bathtub drowning mortality with social welfare expenditure in 2020. SMR refers to the prefecture-specific age-standardized mortality ratio for deaths classified under the International Classification of Diseases 10^th^ revision code W65 occurring at home.

Figure 3 illustrates the association between prefecture-specific SMR and social welfare expenditure for older adults per individual aged ≥65 years in 2020. A negative association between the two factors was observed in prefectures that now have or previously had cities with a medical examiner system, although this trend was not consistent across all prefectures. This trend was also observed in 2019 (eFigure 3). In the linear regression models, none of the demographic, social, lifestyle, environmental, and diagnostic factors measured in 2020 or 2019 were significantly associated with the SMR in the corresponding year (Table 1 and eTable 1).

**Table 1.** Association between prefecture-level representative values and SMR^a^ of drowning and submersion in bath in 2020

Independent variables	*N*	β^b^	(95% CI)	*P* value
Demographic factors				
Population density, people/km^2^	47	−0.01	(−0.07 to 0.05)	0.754
Proportion of elderly single-person households, %	47	−0.07	(−0.24 to 0.09)	0.361
Proportion of individuals aged ≥65 years living alone (%)	47	−0.13	(−0.29 to 0.03)	0.121
Social and lifestyle factors				
Social welfare expense for older adults per people aged ≥65 years, JPY/person	47	−0.11	(−0.27 to 0.05)	0.185
Proportion of employed people aged ≥65 years, %	47	0.05	(−0.12 to 0.21)	0.557
Annual alcohol consumption per capita, kiloliter/person	47	−0.03	(−0.19 to 0.14)	0.726
Environmental factors				
Mean minimum daily temperature in the coldest month, °C	47	−0.08	(−0.24 to 0.08)	0.317
Mean maximum daily temperature in the hottest month, °C	47	−0.02	(−0.18 to 0.15)	0.819
Number of days with snowfalls, days	47	−0.004	(−0.07 to 0.06)	0.893
Mean altitude of prefectural capital, meters	47	−0.002	(−0.06 to 0.06)	0.949
Number of hospitals per 100,000 individuals aged ≥65 years	47	−0.03	(−0.20 to 0.13)	0.680
Diagnostic factors				
Proportion of judicial autopsy, %	47	0.04	(−0.02 to 0.09)	0.212
Proportion of other forms of autopsy, %	47	0.02	(−0.01 to 0.04)	0.184

CI, confidence interval; ICD, International Classification of Diseases; SMR, standardized mortality ratio.

^a^SMR refers to the prefecture-specific age-standardized mortality ratio for deaths classified under the ICD-10 code W65 occurring at home.

^b^The regression coefficients represent a 1 decile increase in population density, the number of days with snowfall, and the mean altitude of the prefectural capital, and a 1 standard deviation increase in the other independent variables.

ICCs for SMRs and the number of public baths, senior welfare centers, daycare service users, home bathing service users, nursing and medical facilities, and geriatric health service facilities were 0.79 (95% confidence interval [CI], 0.71–0.85), 0.96 (95% CI, 0.94–0.97), 0.93 (95% CI, 0.90–0.96), 0.67 (95% CI, 0.58–0.77), 0.84 (95% CI, 0.78–0.89), 0.73 (95% CI, 0.64–0.81), and 0.88 (95% CI, 0.84–0.92), respectively. The number of senior welfare centers, daycare service users, nursing and medical facilities, and geriatric health service facilities were negatively associated with SMR (Table 2). Notably, 1 SD increase in the number of senior welfare centers, daycare service users, nursing and medical facilities, and geriatric health services facilities was significantly associated with a decrease in SMR of 0.07 (95% CI, 0.02–0.11), 0.03 (95% CI, 0.01–0.05), 0.07 (95% CI, 0.04–0.10), and 0.09 (95% CI, 0.05–0.13), respectively, after adjusting for demographic factors (population density [decile]), socioeconomic factors (social welfare expense for individuals aged ≥65 years per capita [JPY/person]), lifestyle factors (annual alcohol consumption per capita [kiloliter/person]), and environmental factors (the mean of lowest daytime outdoor temperature in the coldest month [°C], number of days with snowfalls [decile], and number of hospitals per 10,000 individuals aged ≥65 years). These associations remained significant in the sensitivity analyses excluding data from 2020 (eTable 2).

**Table 2.** Association between bath-related factors and SMR^a^ of drowning and submersion in bath using mixed models

Independent variables	Number of prefectures	Number of observations^b^	β	(95% CI)^c^	*P* value	Adjusted β	(95% CI)^d^	*P* value
Number of Community Wellness Facilities								
Public Baths	47	1,222	0.01	(−0.02 to 0.04)	0.417	−0.01	(−0.06 to 0.04)	0.690
Senior Welfare Centers	47	846	−0.04	(−0.07 to −0.01)	0.011	−0.07	(−0.11 to −0.02)	0.004
Number of Nursing Care Services								
Daycare Service Users	47	987	−0.03	(−0.05 to −0.01)	0.006	−0.03	(−0.05 to −0.01)	0.005
Home Bathing Service Users	47	987	−0.01	(−0.04 to 0.01)	0.341	−0.01	(−0.04 to 0.02)	0.610
Nursing and Medical Facilities	47	987	−0.04	(−0.06 to −0.02)	<0.001	−0.07	(−0.10 to −0.04)	<0.001
Geriatric Health Services Facilities	47	1,128	−0.07	(−0.10 to −0.03)	<0.001	−0.09	(−0.13 to −0.05)	<0.001

CI, confidence interval; ICD, International Classification of Diseases; SMR, standardized mortality ratio.

All independent variables were calculated per 100,000 individuals aged ≥65 years.

^a^SMR refers to the prefecture-specific standardized mortality ratio for deaths classified under ICD-10 code W65, occurring at home.

^b^The data availability periods for the independent variables were as follows: public baths (1995–2020, 26 years), senior welfare centers (2000–2017, 18 years), daycare service users (2000–2020, 21 years), home bathing service users (2000–2020, 21 years), nursing and medical facilities (2000–2020, 21 years), and geriatric health services facilities (1997–2020, 24 years).

^c^Regression coefficients correspond to a 1 standard deviation increase in the independent variables.

^d^Adjusted for population density [decile], social welfare expense for individuals aged ≥65 years [JPY/person], annual alcohol consumption per capita [kiloliter/person], and environmental factors (the mean of lowest daytime outdoor temperature in the coldest month [°C], number of days with snowfalls [decile], and number of hospitals per 10,000 individuals ≥65 years).

## DISCUSSION

Using census data from death certificates covering 1995 to 2020, this study investigated the patterns of W65-coded mortality occurring in home settings across Japan. Specifically, the analysis addressed temporal trends, including both long-term and monthly fluctuations, as well as the distribution of W65-coded deaths by age group. The observed increase in the AMR for W65 over the past 2 decades, which coincides with the rising number of W65-coded deaths, suggests that the increase in W65-coded deaths during this period cannot be solely attributed to Japan’s demographic aging. To our knowledge, this is the first comprehensive epidemiological study to use a complete dataset for accidental drowning and submersion while in a bathtub in Japan. Furthermore, our findings demonstrate a significant association between increases in nursing care facilities and users, particularly those related to bathing practices, and reductions in W65-coded mortality. This relationship highlights important considerations for developing preventive strategies aimed at reducing bath-related deaths.

Our findings are consistent with those of previous studies in terms of the location, age distribution, and monthly trends of deaths. The proportion of bath-related deaths occurring at home was reported to be 94.3% and 85.2% in previous studies based on autopsied cases from the Tokyo Medical Examiner’s Office (*N* = 3,289) and inquest records from Kagoshima Prefecture (*N* = 2,689),^15^^,^^17^ respectively, which is in line with our findings on W65-coded deaths (90.0%). In both previous studies, the age group with the highest number of bath-related deaths was 80–89 years, which is consistent with our results when age groups were categorized into 10-year intervals. However, the age group of 75–79 years ranked second when using 5-year intervals (Figure 1C), indicating that individuals younger than the average life expectancy of Japan are also at a significant risk of W65-coded deaths. Additionally, January and December were identified as the months with the highest and second-highest numbers of bath-related deaths, respectively, in both previous studies, which is consistent with our findings. Thus, the patterns of bath-related deaths, which encompass intrinsic and extrinsic conditions in their definition, closely resembled those observed in W65-coded deaths.

The inverse association observed between the number of nursing care facility users and the lower W65-coded mortality rate can be attributed to the supervised bathing provided in these settings. Daycare services offer supervised bathing environments overseen by care workers or nurses. Similarly, nursing and medical facilities, along with geriatric health service centers, have completely replaced home bathing. In contrast, the number of home bathing service users was not significantly associated with SMR, potentially because of the relatively small number of users. In 2020, the number of home bathing service users was approximately one-twentieth of that of daycare service users across Japan.^18^ Furthermore, the lack of a significant association between the number of public baths and W65-coded mortality may be explained by the fact that some public baths do not provide accessible baths for older adults or offer health education at senior welfare centers.

Although supervised bathing or reduced in-home bathing may help reduce bath-related deaths among older adults with limited physical abilities, several practical challenges could hinder its implementation. Budgetary constraints may restrict the expansion of daycare centers and nursing facilities that provide assisted bathing. Thus, encouraging daycare service users to bathe during service hours within existing facilities may be a more feasible approach. Additionally, long-term bathing habits and privacy concerns may discourage behavioral changes. Therefore, to explore the potential role of supervised bathing in reducing mortality, identifying high-risk populations and circumstances would be essential to guide targeted recommendations before implementing broader preventive measures.

Possible reasons for the inability to identify factors contributing to prefectural SMR differences include diagnostic uncertainty, unmeasured variables, and small sample size (*N* = 47). Bath-related deaths, even those with signs of water inhalation, are not classified as W65 if the examiner registers other conditions that cause drowning in bathtubs, such as cardiovascular disease and stroke. Previous reports have highlighted the personal preferences of medical examiners, police surgeons, and general practitioners in registering drowning deaths as opposed to cardiovascular deaths.^22^ These individual preferences may be more pronounced in regions without the medical examiner system, where general practitioners are requested to perform postmortem examinations.^23^ This could lead to between-prefecture variations in the diagnosis of W65-coded deaths, which may obscure the factors contributing to such diagnoses.

Another possible reason for the failure to identify factors associated with the prefectural SMR may be the omission of housing factors, such as bathroom and living room temperatures, or the proportion of homes with heat insulation. A previous study suggested that colder indoor temperatures may contribute to more pronounced hemodynamic changes during home bathing,^11^ potentially leading to loss of consciousness and subsequent drowning. These factors could explain the relatively lower SMR observed in the northern prefectures. For instance, in Hokkaido Prefecture, the outdoor temperature is exceedingly cold, while the indoor temperature is higher than that of other prefectures during winter.^24^ Additionally, the limited sample size hinders the identification of factors contributing to prefectural differences in SMR. Similarly, the observed association between higher social welfare expenses and lower prefectural SMR (Figure 3A) may not be conclusive.

The strengths of this study include its large sample size and generalizability based on a complete survey of W65-coded deaths across Japan from 1995 to 2020. However, this study had several limitations. First, as this is an ecological study using aggregate data, individual-level relationships cannot be inferred. Second, the cross-sectional design limits causal inferences. Third, we were unable to include deaths that occurred in bathtubs but were classified under other conditions, such as cardiovascular disease or stroke, due to the Japanese death certificate format, which records the location as “home” rather than “bathroom.” Nevertheless, the age and monthly distribution of the W65-coded deaths were consistent with those of previous studies involving a large number of autopsy cases and inquest records. Finally, residual confounding from the unmeasured variables cannot be ruled out. Although we adjusted for age distribution, socioeconomic status, climate, population density, and other factors, we lacked data on regional differences in bathing practices, such as the use of showers versus hot tubs, which may affect both the risk of bath-related deaths and the demand for nursing care services.

### Conclusions

We demonstrated the demographic, temporal, and regional trends in bathtub drowning and submersion deaths using complete data from death certificates. Our findings revealed a negative association between the number of nursing care facilities and users and the SMR for drowning and submersion while in bathtubs at the prefecture level. These results provide a foundation for interventional studies to evaluate the impact of supervised bathing or the avoidance of in-home bathing among older adults on bath-related mortality.

## SUPPLEMENTARY MATERIAL

eTable 1. Association between Prefecture-Level Representative Values and SMR^a^ of Drowning and Submersion in Bath in 2019

eTable 2. Association between Bath–Related Factors and SMR^a^ of Drowning and Submersion in Bath Using Mixed Models (1995–2019)

eFigure 1. SMR of drowning and submersion in bathtubs by prefecture on the Japanese map in 2019

eFigure 2. Five-year changes in age-standardized SMR of accidental drowning and submersion in bathtubs by prefecture (1995–2020)

eFigure 3. Association of bathtub drowning mortality with social welfare expenditure in 2019
