# Supplementary material for: Drowning and Submersion Deaths in Bathtubs and Associated Factors: A Descriptive and Ecological Study in Japan, 1995–2020
Source: J Epidemiol. 2025 Nov 5;35(11):482–9. doi: 10.2188/jea.JE20250032 (PMC12527407; doi:10.2188/jea.JE20250032)
Supplement: Supplementary file 1 [file je-35-482-s001.pdf]

eTable 1. Association between Prefecture-Level Representative Values and SMR[a] of Drowning and Submersion in Bath in 2019

Independent variables	N	β	(95% CI)	*P* value
Demographic factors				
Population density, people/km²	47	−0.02	(−0.19 to 0.14)	0.785
Social and lifestyle factors				
Social welfare expense for older adults per people aged ≥65 years, JPY/person	47	−0.11	(−0.27 to 0.05)	0.182
Proportion of employed people aged ≥65 years, %	47	0.004	(−0.16 to 0.17)	0.956
Annual alcohol consumption per capita, kiloliter/person	47	−0.02	(−0.18 to 0.15)	0.847
Environmental factors				
Outdoor temperature in the coldest month, °C	47	−0.07	(−0.23 to 0.09)	0.397
Outdoor temperature in the hottest month, °C	47	−0.01	(−0.17 to 0.15)	0.917
Number of days with snowfalls, days	47	0.01	(−0.15 to 0.18)	0.874
Mean altitude of prefectural capital, meters	47	−0.03	(−0.19 to 0.13)	0.712
Number of hospitals per 100,000 individuals aged ≥65 years	47	−0.07	(−0.23 to 0.09)	0.373
Diagnostic factors				
Proportion of judicial autopsy, %	47	0.11	(−0.05 to 0.27)	0.155
Proportion of other forms of autopsy, %	47	0.10	(−0.06 to 0.26)	0.229

CI, confidence interval; ICD, International Classification of Diseases; SMR, standardized mortality ratio.

The regression coefficients represent a 1 decile increase in population density, the number of days with snowfall, and the mean altitude of the prefectural capital, and a 1 standard deviation increase in the other independent variables.

[a] SMR refers to the prefecture-specific age-standardized mortality ratio for deaths classified under the ICD-10 code W65 occurring at home.

eTable 2. Association between Bath−Related Factors and SMR[a] of Drowning and Submersion in Bath Using Mixed Models (1995–2019)

Independent variables	Number of prefectures	Number of observations [b]	β	(95% CI) [c]	*P* value	Adjusted β	(95% CI) [d]	*P* value
Number of Community Wellness Facilities								
Public Baths	47	1,175	−0.002	(−0.03 to 0.02)	0.849	−0.01	(−0.06 to 0.04)	0.772
Senior Welfare Centers	47	846	−0.04	(−0.07 to −0.01)	0.011	−0.07	(−0.11 to 0.02)	0.004
Number of Nursing Care Services								
Daycare Service Users	47	940	−0.03	(−0.05 to −0.01)	0.002	−0.03	(−0.05 to −0.01)	0.009
Home Bathing Service Users	47	940	−0.003	(−0.03 to 0.02)	0.836	0.01	(−0.02 to 0.05)	0.539
Nursing and Medical Facilities	47	940	−0.04	(−0.06 to −0.02)	<0.001	−0.07	(−0.10 to −0.03)	<0.001
Geriatric Health Services Facilities	47	1,081	−0.07	(−0.10 to −0.03)	<0.001	−0.08	(−0.12 to −0.04)	<0.001

CI, confidence interval; ICD, International Classification of Diseases; SMR, standardized mortality ratio.

All independent variables were calculated per 100,000 individuals aged 65 years and older.

[a] SMR refers to the prefecture-specific standardized mortality ratio for deaths classified under ICD−10 code W65, occurring at home.

[b] The data availability periods for the independent variables were as follows: public baths (1995–2019, 25 years), senior welfare centers (2000–2017, 18 years), daycare service users (2000–2019, 20 years), home bathing service users (2000–2019, 20 years), nursing and medical facilities (2000–2019, 20 years), geriatric health services facilities (1997–2019, 23 years).

[c] Regression coefficients correspond to a 1 standard deviation increase in the independent variables.

[d] Adjusted for population density [decile], social welfare expense for individuals aged ≥65 years [JPY/person], annual alcohol consumption per capita [kiloliter/person], and environmental factors (the mean of lowest daytime outdoor temperature in the coldest month [°C], number of days with snowfalls [decile], and number of hospitals per 100,000 individuals aged ≥65 years).

eFigure 1. SMR of drowning and submersion in bathtubs by prefecture on the Japanese map in 2019

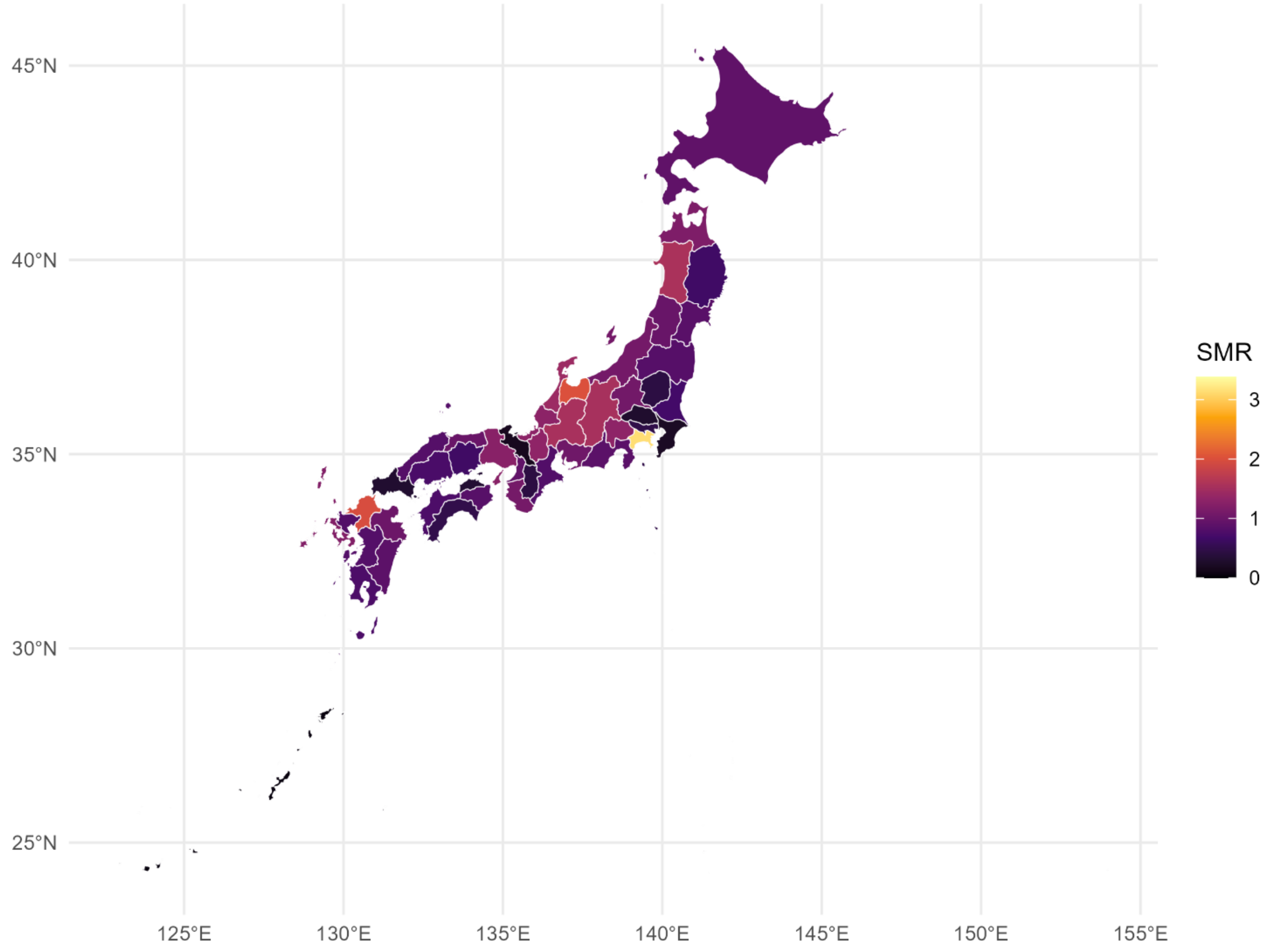

SMR refers to the prefecture-specific age-standardized mortality ratio for deaths classified under the International Classification of Diseases 10th revision code W65 occurring at home.

eFigure 2. Five-year changes in age-standardized SMR of accidental drowning and submersion in bathtubs by prefecture (1995–2020)

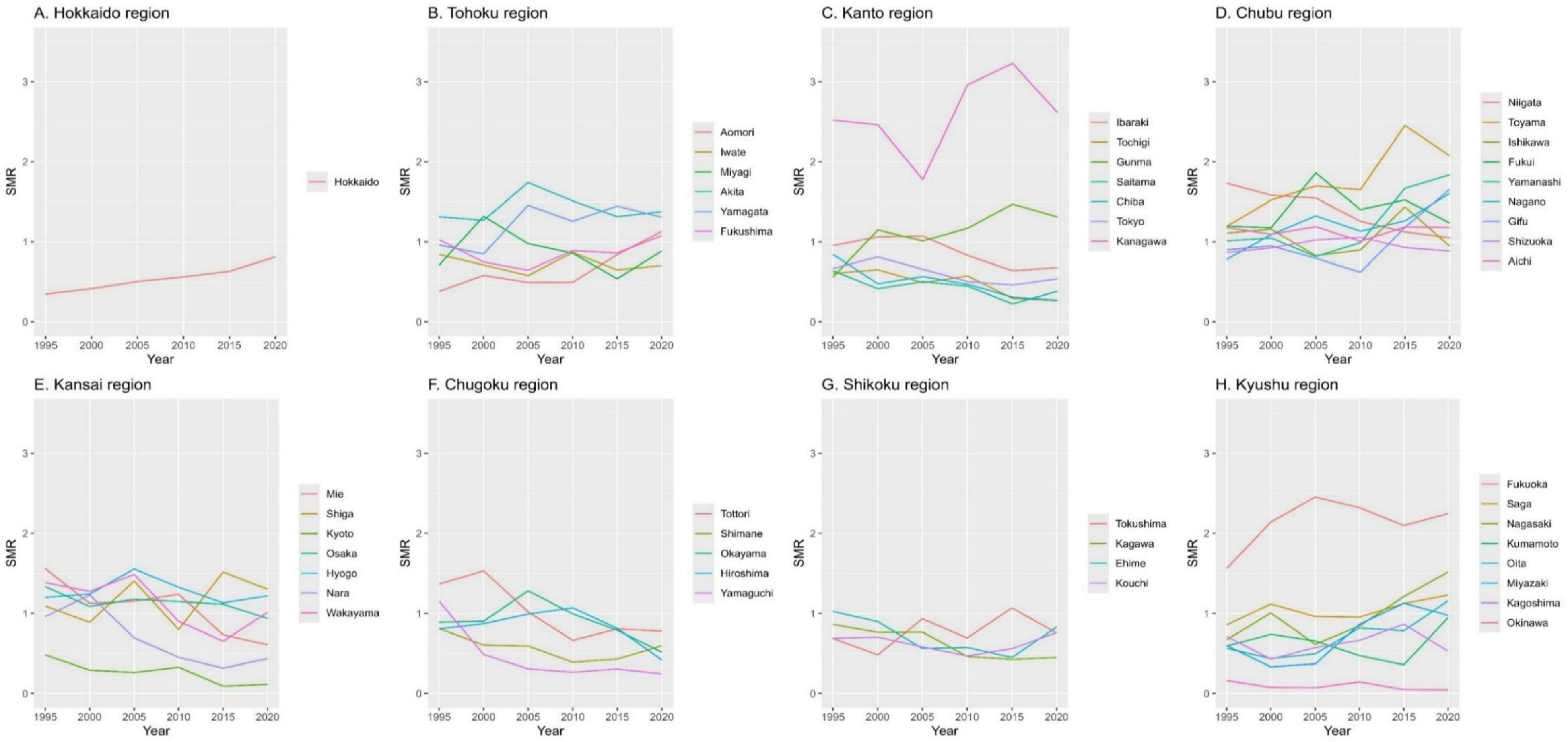

SMR refers to the prefecture-specific age-standardized mortality ratio for deaths classified under the International Classification of Diseases 10th revision code W65 that occurred at home.

eFigure 3. Association of bathtub drowning mortality with social welfare expenditure in 2019

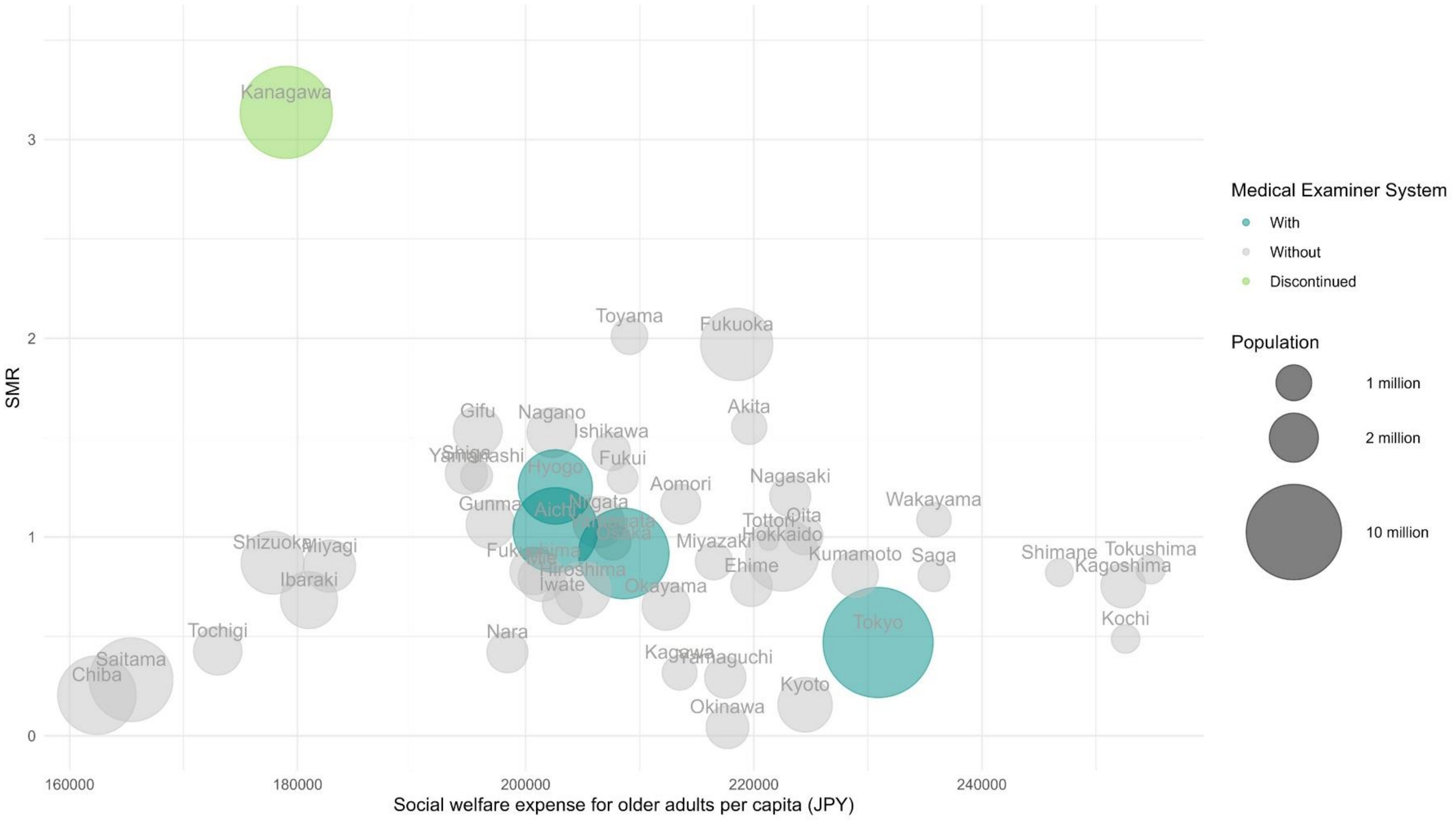

SMR refers to the prefecture-specific age-standardized mortality ratio for deaths classified under the International Classification of Diseases 10th revision code W65 occurring at home.
